# Targeting USP7-Mediated Deubiquitination of MDM2/MDMX-p53 Pathway for Cancer Therapy: Are We There Yet?

**DOI:** 10.3389/fcell.2020.00233

**Published:** 2020-04-02

**Authors:** Si-Min Qi, Gang Cheng, Xiang-Dong Cheng, Zhiyuan Xu, Beihua Xu, Wei-Dong Zhang, Jiang-Jiang Qin

**Affiliations:** ^1^College of Pharmaceutical Sciences, Zhejiang Chinese Medical University, Hangzhou, China; ^2^Institute of Cancer and Basic Medicine, Chinese Academy of Sciences, Cancer Hospital of the University of Chinese Academy of Sciences, Zhejiang Cancer Hospital, Hangzhou, China; ^3^School of Pharmacy, Naval Medical University, Shanghai, China; ^4^Institute of Interdisciplinary Integrative Medicine Research, Shanghai University of Traditional Chinese Medicine, Shanghai, China

**Keywords:** USP7, deubiquitination, p53, MDM2, MDMX, small-molecule inhibitor, cancer

## Abstract

The p53 tumor suppressor protein and its major negative regulators MDM2 and MDMX oncoproteins form the MDM2/MDMX-p53 circuitry, which plays critical roles in regulating cancer cell growth, proliferation, cell cycle progression, apoptosis, senescence, angiogenesis, and immune response. Recent studies have shown that the stabilities of p53, MDM2, and MDMX are tightly controlled by the ubiquitin-proteasome system. Ubiquitin specific protease 7 (USP7), one of the most studied deubiquitinating enzymes plays a crucial role in protecting MDM2 and MDMX from ubiquitination-mediated proteasomal degradation. USP7 is overexpressed in human cancers and contributes to cancer initiation and progression. USP7 inhibition promotes the degradation of MDM2 and MDMX, activates the p53 signaling, and causes cell cycle arrest and apoptosis, making USP7 a potential target for cancer therapy. Several small-molecule inhibitors of USP7 have been developed and shown promising efficacy in preclinical settings. In the present review, we focus on recent advances in the understanding of the USP7-MDM2/MDMX-p53 network in human cancers as well as the discovery and development of USP7 inhibitors for cancer therapy.

## Introduction

Ubiquitination refers to the cellular process by which ubiquitin specifically modifies a target protein under the cascade catalysis of the E1 ubiquitin-activating enzymes, the E2 ubiquitin-conjugating enzymes, and the E3 ubiquitin ligases (Zheng and Shabek, 2017; Senft et al., 2018). The polyubiquitinated proteins are further recognized and rapidly degraded into small peptides by the 26S proteasome (Zheng and Shabek, 2017; Senft et al., 2018). Protein ubiquitination is a common post-translational modification and plays a critical role in many cellular processes, such as apoptosis, cell cycle progression, DNA damage response, and membrane transport by causing the degradation of various regulatory proteins (Senft et al., 2018). Conversely, deubiquitination is the process that removes ubiquitin from the target proteins by the deubiquitinases (DUBs), which can specifically disassemble the ubiquitin-protein conjugates by cleaving the isopeptide bonds formed between the C-terminus of ubiquitin and the target proteins and protect them from degradation and inactivation (Harrigan et al., 2018; Rawat et al., 2019). Therefore, the ubiquitination-deubiquitination balance acts as a critical and powerful way to regulate protein turnover and functions and has been demonstrated as a promising target for developing therapeutic agents for several diseases, including cancer.

Ubiquitin-specific protease 7 (USP7), also named as herpesvirus-associated ubiquitin-specific protease (HAUSP) is one of the most investigated DUBs and has been associated with the initiation and progression of various cancers (Masuya et al., 2006; Su et al., 2018). USP7 has a broad range of substrates, such as the tumor suppressors p53 (Li et al., 2002) and PTEN (Phosphatase and tensin homolog) (Song et al., 2008) and the transcription factor FOXO4 (Forkhead box protein O4) (van der Horst et al., 2006), most of which are related to tumor suppression, DNA repair, and immune response (Pozhidaeva and Bezsonova, 2019). USP7 is also capable of deubiquitinating and stabilizing MDM2 (Murine double minute 2) and MDMX (Murine double minute X, also known as MDM4) oncoproteins, which are the major negative regulators of the p53 tumor suppressor (Sheng et al., 2006; Sarkari et al., 2010). Herein, targeting USP7 for destabilizing MDM2/MDMX and activating p53 has recently been proposed as a promising strategy for cancer therapy, and several USP7 inhibitors have been developed and shown promising efficacy in preclinical cancer models *in vitro* and *in vivo* (Zhou et al., 2018).

Due to the multifaceted nature of USP7 in various diseases, its structure, enzymatic activity, substrates, regulation, disease relevance, and inhibitors have been extensively reviewed recently (Bhattacharya et al., 2018; Wu et al., 2018; Zhou et al., 2018; Pozhidaeva and Bezsonova, 2019; Rawat et al., 2019). In the present review, we focus on the USP7-MDM2/MDMX-p53 network in human cancers as well as the current targeting strategies and small-molecule inhibitors. We also propose new targeting approaches that may lead to more specific and effective inhibitors for treating human cancers.

## The USP7-MDM2/MDMX-p53 Network

### The Protein Structures of USP7, MDM2, MDMX, and p53

**FIGURE 1:**
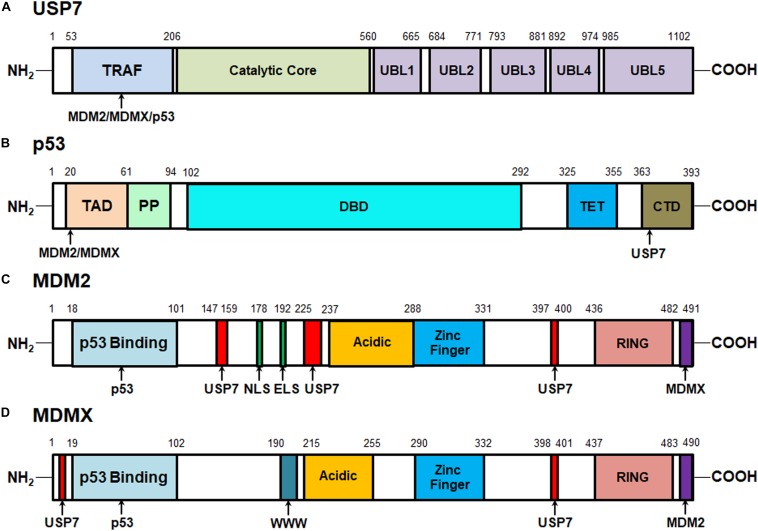
Structures of USP7, MDM2, MDMX, and p53 proteins and their interactive sites. **(A)** Structure of USP7 and the binding sites of MDM2, MDMX, and p53. **(B)** Structure of p53 and the MDM2-, MDMX-, and USP7-binding sites. **(C)** Structure of MDM2 and the p53-, MDMX-, and USP7-binding sites. **(D)** Structure of MDMX and the p53-, MDM2-, and USP7-binding sites.

The human *USP7* gene is located on chromosome 16p13.2 and was initially identified as a new member of the ubiquitin-specific protease (USP) family in 1997 (Everett et al., 1997). The USP7 protein consists of 1102 amino acids that are distributed in three major domains, including the N-terminal tumor necrosis factor receptor-associated factor (TRAF) domain (amino acids 53–206), a central catalytic domain (amino acids 208–560), and the C-terminal tandem ubiquitin-like (Ubl) domain (UBL1-5, amino acids 560–1102) (Figure 1A). Among these domains, TRAF is critical for the binding of USP7 to its substrates, including MDM2, MDMX, and p53 via P/AxxS motifs (Hu et al., 2002, 2006; Saridakis et al., 2005; Sheng et al., 2006; Sarkari et al., 2010; Rouge et al., 2016). It has also been found that the nuclear localization of USP7 is partially dependent on the TRAF domain through the production of USP7 domain deletion mutants (Fernandez-Montalvan et al., 2007; Tavana and Gu, 2017).

The catalytic core of USP7 contains three distinct regions, Thumb, Palm, and Fingers, which form a unique binding pocket for ubiquitin. When ubiquitin binds, the catalytic core undergoes conformational changes, rearranging the catalytic ternary residues to adjacent positions and allowing ubiquitin catalysis (Hu et al., 2002). Mutations in the catalytic core region have been shown to significantly reduce USP7 activation (Faesen et al., 2011). Therefore, this catalytic domain is mainly responsible for the binding of USP7 with ubiquitin and the deubiquitination of the substrates (Hu et al., 2002). However, the weak catalytic activity of the separated catalytic core indicates that other regions are helpful to improve the efficiency of the ubiquitin catalytic reaction.

The C-terminal Ubl domains, which are ordered in a 2-1-2 manner (UBL1/2, UBL3, UBL4/5), contribute to the binding ability of USP7 to specific substrates and its deubiquitinating activity (Faesen et al., 2011; Kim et al., 2016). *In vitro* tests of mutants with different deletion regions have shown that the deletion of C-terminal strongly reduces the deubiquitination activity of USP7. Most of the catalytic activity of USP7 is reconstituted by adding UBL4/5 and 19 amino acid C-terminal tails, which suggests that they play an important role in this specific domain. Further mechanism analyses have found that UBL4/5 directly interacts and synergizes with the switch circuit in the catalytic field, which promotes the conformational change and thus increases the USP7 affinity to ubiquitin (Faesen et al., 2011). Therefore, all three domains are important for the recognition of the substrates by USP7 and the subsequent removal of ubiquitin from the target proteins (Tavana and Gu, 2017).

One of the most important USP7-interactive proteins is the p53 tumor suppressor (Saridakis et al., 2005). As shown in Figure 1B, p53 has 393 amino acid residues that can be subdivided into five domains: the N-terminal transactivation domains (TAD) that are responsible for its binding to the p53-binding sites on MDM2 and MDMX, a proline-rich region (PP) that contains five PxxP motifs and is essential for inducing apoptosis, a DNA-binding domain (DBD), a tetramerization domain (TET), and a C-terminal domain (CTD) that is critical for the binding of p53 to the TRAF domain of USP7 (Nag et al., 2013; Laptenko and Prives, 2017). As disclosed by biochemical and structural studies, the p53 CTD includes the amino acid residues ^359^PGGSRAHSS^367^ that harbor two distinct USP7-binding sites while Ser362 and Ser367 are essential for the USP7-p53 binding (Sheng et al., 2006).

The oncoproteins MDM2 and MDMX are not only the major negative regulators of p53 but also the well-characterized substrates of USP7 (Sarkari et al., 2010; Nag et al., 2013; Karni-Schmidt et al., 2016; Wang et al., 2019b). MDM2 and MDMX include 491 and 490 amino acid residues, respectively and share a great structural similarity (Nag et al., 2013; Karni-Schmidt et al., 2016). As depicted in Figures 1C,D, both MDM2 and MDMX proteins contain an N-terminal p53-binding domain, a central acidic domain that contributes to p53 ubiquitination, a zinc finger domain, and a C-terminal RING domain which is critical for MDM2’s E3 ligase activity and the formation of MDM2-MDMX heterodimers. Besides, MDM2 has the nuclear localization signal (NLS) and export localization signal (ELS) sequences but MDMX only has a unique WWW motif without NLS and ELS. The recent biochemical and structural analyses have characterized four USP7-binding sites on MDM2, including ^147^PSSSHLVSRPSTS^159^, ^226^AGVS^229^, and ^397^PSTS^400^, and Ser150 and Ser229 are critical for the MDM2-USP7 binding (Hu et al., 2006; Sheng et al., 2006; Sarkari et al., 2010). Also, two P/AxxS motifs of MDMX, ^8^AQCS^11^ and ^398^AHSS^401^ have been recognized and validated as the USP7-binding sites (Sarkari et al., 2010). These studies not only lead to a better understanding of the molecular recognition mechanisms of p53, MDM2, and MDMX by USP7 but also provide a viable option for developing new protein-protein interaction inhibitors.

### The Regulation of MDM2/MDMX-p53 Circuitry by USP7

**FIGURE 2:**
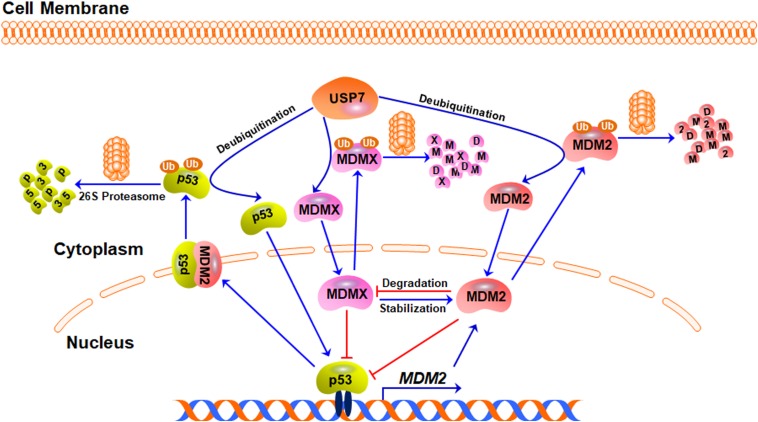
The USP7-MDM2/MDMX-p53 axis. Under stress-free conditions, MDM2, MDMX, and p53 form a negative feedback loop, in which p53 activates MDM2 and increases its expression level whereas MDM2 and MDMX directly bind to p53 and inhibit its transcriptional activity. MDM2 also acts as an E3 ubiquitin ligase and induces the ubiquitination and degradation of p53, MDMX, and MDM2 itself. USP7 directly binds to and stabilizes MDM2, MDMX, and p53 proteins through its deubiquitinating activity.

Although many USP7 substrates have been identified, the best-investigated function of USP7 is still the regulation of the MDM2/MDMX-p53 circuitry (Figure 2). As the major negative regulator of p53, MDM2 can directly bind to p53 and inhibit the transcription of its downstream target genes, including *MDM2* itself, forming a negative feedback loop (Momand et al., 1992; Oliner et al., 1992; Barak et al., 1993; Perry et al., 1993). Importantly, MDM2 not only inhibits p53-mediated transactivation of its target genes but also promotes p53 ubiquitination and degradation by acting as an E3 ubiquitin ligase (Haupt et al., 1997; Honda et al., 1997; Kubbutat et al., 1997). Furthermore, the MDM2 homolog MDMX also binds to p53 and inhibits its transcriptional activity (Shvarts et al., 1996; Shvarts et al., 1997). However, MDMX does not affect the protein stability of p53 because it lacks the E3 ligase activity (Shvarts et al., 1996). MDM2 can also promote the ubiquitination and proteasomal degradation of MDMX and itself (Fang et al., 2000; de Graaf et al., 2003; Pan and Chen, 2003), while MDMX can bind to and stabilize MDM2 protein (Tanimura et al., 1999).

USP7 was initially identified as a p53-interactive protein by mass spectrometry analysis (Li et al., 2002). It has been found that the overexpression of USP7 stabilizes p53 even in the presence of excess MDM2 and the down-regulation of USP7 expression makes the endogenous p53 unstable (Tavana and Gu, 2017). Mechanistically, USP7 interacts with p53 and removes the ubiquitin from p53, causing p53 stabilization (Li et al., 2002). However, it has also been observed that the disruption of the *USP7* gene stabilizes p53 (Cummins et al., 2004), which has been attributed to USP7-mediated MDM2 deubiquitination and stabilization (Li et al., 2004). Compared with p53, the binding between MDM2 and USP7 is much stronger (Hu et al., 2006; Sheng et al., 2006). USP7 binds to and stabilizes MDM2 under stress-free conditions, resulting in p53 turnover in an MDM2-dependent manner (Rawat et al., 2019). When the decrease of the USP7 level leads to MDM2 degradation, the available MDM2 pool will be not enough to exert the ubiquitin E3 ligase activity, which leads to the stabilization of p53 (Li et al., 2004). USP7 also deubiquitinates and stabilizes MDMX and the inhibition of USP7 causes MDMX degradation and the indirect activation of p53 (Meulmeester et al., 2005).

Taken together, USP7 is able to remove the ubiquitin from p53, MDM2, and MDMX and keep the stabilities of these proteins by rescuing them from degradation (Figure 2). However, due to the critical negative regulation of MDM2 and MDMX on p53, USP7-mediated stabilization of MDM2 and MDMX also causes p53 inactivation and degradation, which makes a paradoxical role of USP7 in regulating p53.

## The Role of USP7-MDM2/MDMX-p53 Network in Human Cancer and Immune Response

The p53 tumor suppressor plays a critical role in protecting normal cells from transformation and tumorigenesis through transactivation of the genes related to cell growth arrest, apoptosis, and senescence (Nag et al., 2013; Liu et al., 2019a). The MDM2 and MDMX oncogenes have been demonstrated to promote tumor growth and metastasis via both p53-dependent and p53-independent mechanisms (Karni-Schmidt et al., 2016; Wang et al., 2019b). The overexpression and amplification of MDM2 and MDMX and the loss-of-function mutation of p53 are the common and crucial events in the initiation, progression, and metastasis of human cancers, which have been comprehensively discussed recently (Walerych et al., 2015; Hou et al., 2019).

USP7, as a crucial controller of the MDM2/MDMX-p53 circuitry, has shown important functions in various human cancers and its expression and activation are associated with the progression and prognosis of these diseases (Table 1) (Bhattacharya et al., 2018; Zhou et al., 2018). It has been reported that both the protein and mRNA levels of USP7 are highly elevated in multiple myeloma (MM), and the MM patients with higher USP7 levels have significantly poorer survival (Chauhan et al., 2012). A similar role of USP7 has also been found in gliomas, neuroblastoma, and ovarian cancer, and USP7 overexpression is correlated with the disease progression and poor survival of patients (Cheng et al., 2013; Fan et al., 2013; Ma and Yu, 2016). Moreover, the carcinogenic role of USP7 in these types of cancer has been attributed to USP7-mediated deubiquitination and stabilization of MDM2 and MDMX, which has been validated by demonstrating the anticancer activity of USP7 inhibitors *in vitro* and *in vivo* (Chauhan et al., 2012; Fan et al., 2013).

**TABLE 1:** Epidemiological and clinical evidence connecting USP7 and cancer.

Cancer type	Expression	Mechanisms	Clinical outcomes	References
Multiple myeloma	Overexpression	Deubiquitinates and stabilizes MDM2 and MDMX	Worse survival	Chauhan et al., 2012
Gliomas	Overexpression	Deubiquitinates and stabilizes MDM2	Disease progression and worse survival	Cheng et al., 2013
Neuroblastoma	Overexpression	Deubiquitinates and stabilizes MDM2	Worse survival	Fan et al., 2013
Ovarian cancer	Overexpression	Deubiquitinates and stabilizes MDM2	Lymph node metastasis and worse survival	Ma and Yu, 2016
Prostate cancer	Overexpression	PTEN nuclear exclusion	Increased tumor aggressiveness	Song et al., 2008
Breast cancer	Overexpression	Deubiquitinates and stabilizes ERα and PHF8		Wang et al., 2016b; Xia et al., 2019
Cervical cancer	Overexpression	Deubiquitinates and stabilizes MDC1	Worse survival	Su et al., 2018
Colorectal cancer	Overexpression	Deubiquitinates and stabilizes β-catenin	Worse survival	An et al., 2017
Non-small cell lung cancer (excluding adenocarcinoma)	Overexpression	Deubiquitinates and stabilizes MDM2	Poor prognosis	Zhao et al., 2015
Non-small cell lung cancer (adenocarcinoma)	Reduced expression	p53-dependent mechanisms	Carcinogenesis and poor prognosis	Masuya et al., 2006

USP7 overexpression has been observed in prostate cancer, which is correlated with the increased aggressiveness of the tumors. Moreover, the high expression level of USP7 is associated with the nuclear exclusion of the tumor suppressor PTEN (Song et al., 2008). USP7 is also overexpressed in breast, cervical, and colorectal cancer, which is positively associated with the worse survival of patients with these diseases (An et al., 2017; Su et al., 2018; Xia et al., 2019). In breast cancer cells, USP7 physically interacts with estrogen receptor α (ERα) and histone demethylase PHF8 and promotes their deubiquitination and stabilization (Wang et al., 2016b; Xia et al., 2019). In cervical cancer cells, USP7 directly binds to the MRN (MRE11-RAD50-NBS1)-MDC1 (mediator of DNA damage checkpoint protein 1) complex and deubiquitinates and stabilizes MDC1, which results in the maintenance of the DNA damage response (DDR) (Su et al., 2018). In colorectal cancer cells, USP7 may regulate the deubiquitination of β-catenin, although the detailed binding mechanisms need to be further investigated (An et al., 2017). Considering that MDM2 and MDMX are highly expressed in prostate, breast, cervical, and colorectal cancer (Cressey et al., 2006; Voruganti et al., 2015; Qin et al., 2016a, b, 2017; Stiasny et al., 2017; Wang et al., 2018a), USP7-mediated stabilization of MDM2 and MDMX may also contribute to the progression of these diseases.

Of note, USP7 is highly expressed in non-small cell lung cancer (NSCLC) and correlated with poor overall survival of patients (Zhao et al., 2015), excluding adenocarcinoma (Masuya et al., 2006). Conversely, NSCLC adenocarcinoma shows a lower expression level of USP7, which is negatively associated with the prognosis of this disease through p53-dependent mechanisms (Masuya et al., 2006), making a controversial and content-dependent role of USP7 in NSCLC. The contradictory effects of USP7 have also been found in the human colon carcinoma xenograft model (Becker et al., 2008). Both USP7 up- and down-regulation have been shown to stabilize and activate p53, thereby inhibiting the cancer cell growth and enhancing radiotherapy *in vitro* and *in vivo*. However, it has also been found that the down-regulation of USP7 reduces the camptothecin-induced mitochondrial translocation of p53, which further causes the resistance of cancer cells to stress-induced apoptosis (Becker et al., 2008).

USP7 has recently been found to promote tumor evasion of the immune system by deubiquitinating and stabilizing its substrates, e.g., Foxp3 (Forkhead box P3) and Tip60 (Tat-interactive protein-60 kDa) that play critical roles in enhancing the immunosuppressive functions of regulatory T cells (Treg) and suppressing tumoricidal effector T cells (Teff) (van Loosdregt et al., 2013; Wang et al., 2016a, 2017a). Wang et al. (2016b) have demonstrated that *USP7* deletion in Treg cells results in the expression alterations of 1,477 genes, notably causing the decreased expression of many transcription factors that play pivotal roles in Treg cell development and stability and the increased expression of genes associated with cell cycle, apoptosis, DNA damage response, and glycolysis pathways. Consequently, *USP7* deletion significantly impairs the suppressive functions of Treg cells *in vitro* and *in vivo*. Therefore, USP7 has recently been proposed as a target of both tumor growth and immune evasion (Wu et al., 2018). Pharmacological modification of USP7 by its small-molecule inhibitors (including P5091 and P0217564) have been examined and shown promising efficacy in abrogating Treg cell functions and promoting antitumor immunity in murine Treg cells *in vitro* and in Treg-dependent syngeneic mouse models *in vivo* (Wang et al., 2016a; Fu et al., 2019). Wang and colleagues have also found that USP7 inhibitor treatment leads to the enhanced accumulation of CD8^+^ T cells in the tumor microenvironment and the reduced accumulation of Foxp3^+^ Treg cells, which cause the increased production of IFNγ and the break of immune tolerance. Therefore, the USP7 inhibitors could favorably target the Foxp3^+^ Treg cells and inhibit their functions, indicating that targeting USP7 may be a promising strategy to break immune tolerance and facilitate immunotherapy in cancer. However, it should be noted that the developmental deletion of *USP7* in Foxp3^+^ Treg cells causes lethal systemic autoimmunity in mice within 4 weeks of birth (Wang et al., 2016a). It is still not clear whether USP7 inhibitor-mediated repression of Treg function impairs host immune responses and induces autoimmunity. Further investigation should be performed in more clinically relevant cancer models *in vitro* and *in vivo*.

## Targeting MDM2/MDMX-p53 Circuitry by USP7 Inhibitors

**FIGURE 3:**
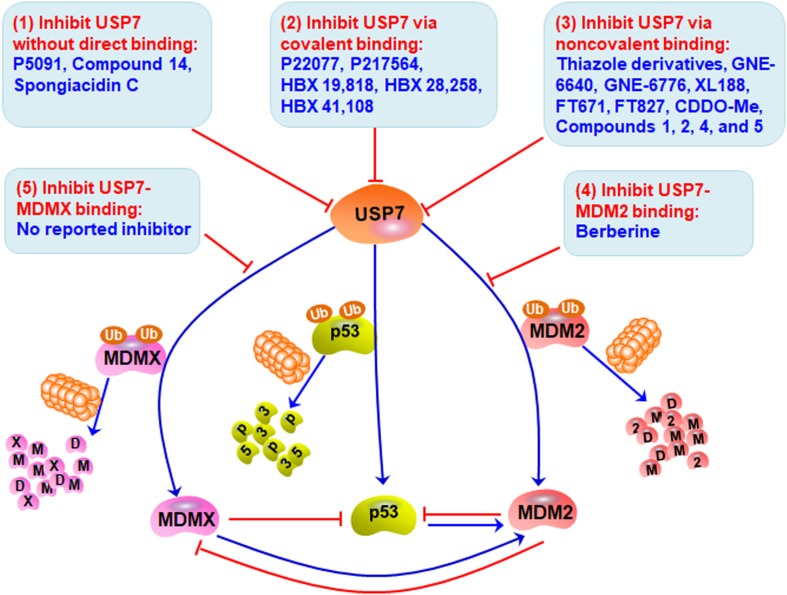
Targeting USP7-MDM2/MDMX-p53 network for cancer therapy. Several small-molecule inhibitors have been developed to broadly inhibit the deubiquitinating activity of USP7 without direct binding (Strategy 1), via covalent binding (Strategy 2), or via non-covalent binding (Strategy 3), therefore inducing the degradation of MDM2 and MDMX and stabilizing and activating p53. These compounds have shown anticancer efficacy *in vitro* and *in vivo*. Directly inhibiting the bindings of USP7 to MDM2 (Strategy 4) and MDMX (Strategy 5) may be examined to develop more safe and effective inhibitors for cancer therapy.

Considering the pivotal role of USP7 in regulating the MDM2/MDMX-p53 pathway in cancer cells, many small-molecule inhibitors have been developed for treating human cancers. The majority of USP7 inhibitors have been found to inhibit its deubiquitinating activity, promote the degradation of MDM2 and MDMX, and activate p53 signaling (Table 2 and Figure 3). All the deubiquitinating activity inhibitors can be classified into three categories based on their molecular mechanisms, i.e., inhibiting USP7 activity (1) without direct binding, (2) via covalent binding, or (3) via non-covalent binding. It has also been reported that direct inhibition of the USP7-MDM2 binding can promote MDM2 ubiquitination and degradation (strategy 4 in Figure 3). Accordingly, direct inhibition of the USP7-MDMX interaction may cause MDMX degradation, but there is no specific inhibitor reported yet (strategy 5 in Figure 3).

**TABLE 2:** Representative USP7 inhibitors and their anticancer activities and mechanisms of action.

Inhibitors	Mechanisms of action	*In vitro* activity	*In vivo* activity	References
**Inhibiting USP7 activity without direct binding**				
P5091	Inhibits USP7 deubiquitinating activity, promotes protein degradation of MDM2, MDMX and β-catenin, and activates p53 and p21	Induces MM cell death, overcomes bortezomib- resistance, and exerts synergistic anti-MM activity in combination with lenalidomide, HDAC inhibitor, and dexamethasone	Inhibits MM xenograft tumor growth, prolongs survival of mice, and blocks angiogenesis in mice, regardless of p53 status	Nicholson and Suresh Kumar, 2011; Chauhan et al., 2012
		Inhibits proliferation and induces apoptosis of CRC cells	Suppresses tumor growth in the HCT116 and CT26 xenograft mouse models	An et al., 2017; Fu et al., 2019
		Suppresses the growth of ovarian cancer cells, causes cell cycle blockage, and induces necrosis and apoptosis	NR	Wang et al., 2017b
		Decreases the viability of Ewing sarcoma cells.	NR	Stolte et al., 2018
Compound 14	Inhibits USP7 deubiquitinating activity and activates p53 and p21	NR	NR	Weinstock et al., 2012
Spongiacidin C	Inhibits USP7 deubiquitinating activity, promotes MDM2 degradation, and activates p53	NR	NR	Yamaguchi et al., 2013
**Inhibiting USP7 activity via covalent binding**				
P22077	Covalently binds to USP7 active site, inhibits its deubiquitinating activity, promotes the degradation of MDM2 and Tip60, and activates p53 and p21	Induces apoptosis and enhances chemosensitivity in NB cells	Inhibits tumor growth in mice bearing IMR-32, SH-SY5Y, and NGP orthotopic xenograft tumors	Altun et al., 2011; Dar et al., 2013; Fan et al., 2013; Pozhidaeva et al., 2017
HBX 19,818	Covalently binds to USP7 active site, inhibits its deubiquitinating activity, promotes MDM2 protein degradation, and activates p53	Inhibits cancer cell proliferation and induces apoptosis and cell cycle arrest at G1 phase	NR	Reverdy et al., 2012
HBX 28,258	Covalently binds to USP7 active site, inhibits its deubiquitinating activity, promotes MDM2 protein degradation, and activates p53	NR	NR	Reverdy et al., 2012
HBX 41,108	Inhibits USP7 deubiquitinating activity and activates p53	Inhibits cancer cell growth and induces apoptotic cell death in a p53-dependent manner	NR	Colland et al., 2009
FT827	Covalently bind to the inactive (apo) form of USP7 at the ubiquitin-binding site	NR	NR	Turnbull et al., 2017
P217564	Covalently binds to USP7 active site, inhibits its deubiquitinating activity, promotes the degradation of MDM2, Tip60, and Foxp3	Impairs suppressive function of Treg cells	Inhibits tumor growth in syngeneic mice bearing TC1 flank tumors	Wang et al., 2016b,2017b
**Inhibiting USP7 activity via non-covalent binding**				
Thiazole derivatives C7 and C19	Non-covalently binds to USP7 and inhibits its deubiquitinating activity	Inhibits cell proliferation, independent of p53	NR	Chen et al., 2017
XL188	Non-covalently binds to USP7 and inhibits its deubiquitinating activity	NR	NR	Lamberto et al., 2017
CDDO-Me	Non-covalently binds to USP7, inhibits its activity, and induces the degradation of MDM2, MDMX, and UHRF1	Inhibits the proliferation of ovarian cancer cells	Suppresses tumor growth in mice bearing HO8910 and SKOV3 xenograft tumors	Qin et al., 2016
GNE-6640 and GNE-6776	Non-covalently binds to USP7 and competitively inhibits ubiquitin-USP7 binding	Inhibit cancer cell viability and enhance the anticancer activity of chemotherapeutics and targeted agents	Inhibits tumor growth in EOL-1 xenograft models	Kategaya et al., 2017
FT671	Non-covalently bind to the inactive (apo) form of USP7 at the ubiquitin-binding site	Inhibits the proliferation of MM.1s cells	Inhibits tumor growth without weight loss or cachexia in an MM.1S xenograft model	Turnbull et al., 2017
Compound 1	Non-covalently binds to USP7 and inhibits its deubiquitinating activity	NR	NR	Gavory et al., 2018
Compound 2	Non-covalently binds to USP7 and inhibits its deubiquitinating activity	NR	NR	Gavory et al., 2018
Compound 4	Non-covalently binds to USP7, inhibits its deubiquitinating activity, promotes the degradation of MDM2, and activates p53 and p21	Inhibits the proliferation of RS4;11 and LNCaP cells	NR	Gavory et al., 2018
Compound 5	Non-covalently binds to USP7 and inhibits its deubiquitinating activity	NR	NR	Gavory et al., 2018
**Directly inhibiting USP7-MDM2 binding**				
Berberine	Disrupts the MDM2-DAXX-USP7 interaction and induces MDM2 self-ubiquitination	Induces apoptosis of ALL cell lines with wild type p53 and overexpressed MDM2	NR	Zhang et al., 2010

*ALL, acute lymphoblastic leukemia; CRC, colorectal cancer; HDAC, histone deacetylase; MDM2, murine double minute 2; MDMX, murine double minute X; MM, multiple myeloma; NB, neuroblastoma; NR, not reported; USP7, ubiquitin specific peptidase 7.*

### Inhibiting USP7 Activity Without Direct Binding

P5091 is the first identified specific USP7 inhibitor through a high-throughput screening from a small-molecule library using a ubiquitin-phospholipase A2 enzyme reporter assay (Nicholson and Suresh Kumar, 2011; Chauhan et al., 2012). It has been found that P5091 potently and selectively inhibits the deubiquitinating activity of USP7 with an IC_50_ value of 4.2 μM, without affecting other DUBs at 100 μM (Chauhan et al., 2012). As expected, P5091 induces the polyubiquitination of MDM2 and MDMX and accelerates their proteasomal degradation in MM cells, thereby activating p53 and its downstream target p21 (Chauhan et al., 2012). It has further been observed that P5091 induces cancer cell death and overcomes chemoresistance in MM cells *in vitro* and in MM xenograft models *in vivo*. Moreover, P5091 has been shown to inhibit cell proliferation and induce cell cycle arrest and apoptosis in colorectal cancer (An et al., 2017; Fu et al., 2019), ovarian cancer (Wang et al., 2017b), and Ewing sarcoma (Stolte et al., 2018) cells *in vitro* and *in vivo*. Besides, P5091 inhibits the Wnt signaling by enhancing the ubiquitination and degradation of β-catenin, suggesting that β-catenin is a potential substrate of USP7 (An et al., 2017).

Considering the important role of USP7 in Treg cell functions, Wang et al. (2016a) have recently demonstrated that P5091 promotes the antitumor immune response by impairing the immunosuppressive activity of Treg cells. Fu et al. (2019) have further evaluated the efficacy and possible molecular mechanisms of P5091 in the colon cancer CT26 xenograft model. The USP7 inhibitor has comparable efficacy versus anti-PD-1 antibody in inhibiting the growth of CT26 xenograft tumors in mice. Mechanism studies have shown that P5091 treatment down-regulates IL-10 expression and upregulates the expression of IFN-γ and TNF-α in tumor tissue at the mRNA level (Fu et al., 2019). Similar changes have been observed in serum from tumor-bearing mice at the protein level. Importantly, P5091 treatment increases IFN-γ expression in both CD4^+^ and CD8^+^ T cells from the spleen of tumor-bearing mice, which suggests that this compound boosts the antitumor immune response (Fu et al., 2019). It has been further found that P5091 down-regulates the expression of Foxp3 in CD4^+^ T cells and decreases the proportion of Treg cells *in vivo*, suggesting an impaired immune tolerance in the tumor microenvironment.

In addition, a new class of P5091 derivatives has recently been designed and synthesized, and a lead compound termed compound 14 has been discovered to inhibit the deubiquitinating activities of both USP7 and USP47 with IC_50_ values of 0.42 and 1.0 μM, respectively (Weinstock et al., 2012). Compound 14 also activates p53 and p21 in HCT116 human colon cancer cells (Weinstock et al., 2012). A pyrrole alkaloid, named spongiacidin C has recently been isolated from the marine sponge *Stylissa massa* and identified as a specific USP7 inhibitor with an IC_50_ of 3.8 μM (Yamaguchi et al., 2013). However, neither compound 14 nor spongiacidin C are evaluated for their anticancer activity yet.

### Inhibiting USP7 Activity via Covalent Binding

The medicinal chemistry optimization of P5091 leads to the identification of a USP7/USP47 dual inhibitor termed P22077 (Altun et al., 2011). In comparison to P5091, P22077 shows low inhibitory effects on the deubiquitinating activity with effective concentrations at 15–45 μM (Altun et al., 2011). The compound has further been shown to promote MDM2 degradation and activate p53 and p21 (Altun et al., 2011). Pozhidaeva et al. (2017) have recently demonstrated that P22077 covalently modifies cysteine 223 (Cys223) in the catalytic core of USP7 and induces conformational changes in the active site, resulting in the inhibition of its enzymatic activity. P22077 also induces apoptosis and enhances chemosensitivity in neuroblastoma (NB) cells *in vitro* (Fan et al., 2013). Its *in vivo* efficacy has been demonstrated in mice bearing IMR-32, SH-SY5Y, and NGP orthotopic xenograft tumors (Fan et al., 2013). Based on the observation that P22077 promotes the ubiquitination and degradation of Tip60, this protein has been identified as a substrate of USP7 and has been found to contribute to P22077-mediated cytotoxicity (Dar et al., 2013).

The USP7 inhibitors P22077 and compound 14 have shown unexpected inhibition on USP47 (Altun et al., 2011; Weinstock et al., 2012). To identify more specific USP7 inhibitors, Reverdy et al. (2012) have developed a high-throughput screening assay using an optimized USP substrate ubiquitin C-terminal 7-amido-4-methylcoumarin, which has led to the discovery of a novel class of USP7 inhibitors from a chemically diverse library of small molecules. The lead compounds HBX 19,818 and HBX 28,258 can specifically and selectively inhibit USP7 activity with IC_50_ values of 28.1 and 22.6 μM, respectively, without apparent effects on other tested deubiquitinating enzymes (Reverdy et al., 2012). Importantly, this class of compounds can covalently form a bond with the Cys223 located in the catalytic core of USP7, thereby selectively causing its inactivation as well as MDM2 degradation and p53 stabilization and activation (Reverdy et al., 2012). HBX 19,818 has further been shown to inhibit cell proliferation and induce G1 arrest and apoptosis in HCT116 cells. It has also been found that HBX 19,818 inhibits cancer cell viability in a p53-independent manner, which emphasizes the critical role of the p53-independent functions of MDM2 and MDMX in cancer cells (Qin et al., 2018; Wang et al., 2019b). However, both HBX 19,818 and HBX 28,258 have not been evaluated in any animal models, and further investigation for their *in vivo* efficacy and safety should be performed.

Further optimization of HBX 19,818 and HBX 28,258 has led to a more potent and specific inhibitor named HBX41,108, which inhibits USP7 activity with an IC_50_ of 424 nM (Colland et al., 2009). This new USP7 inhibitor has been found to significantly reverse the USP7-mediated MDM2 stabilization and increase the expression of p53 and p21 in a dose-dependent manner. The molecular modeling and docking studies have been performed and the results suggest that HBX41,108 may covalently bind to the ubiquitin-binding sites on USP7 and allosterically inhibit its catalytic activity (Colland et al., 2009). Importantly, HBX41,108 can significantly inhibit cell proliferation and induce apoptosis in HCT116 cells at submicromolar concentrations (Colland et al., 2009). Another high-throughput screening of a library containing ∼500K compounds at FORMA Therapeutics has recently been performed by using a ubiquitin-rhodamine assay, and a covalent inhibitor FT827 has been identified (Turnbull et al., 2017). Similar to HBX 19,818 and HBX 28,258, FT827 binds to the catalytic domain of USP7 (*K*_*D*_ = 7.8 μM) by covalently modifying Cys223 of this domain and inhibits the enzymatic activity (Turnbull et al., 2017). However, the *in vivo* efficacy, toxicology, and pharmacokinetics of HBX41,108 and FT827 are still unknown yet, which are critical for further developing these compounds as anticancer agents.

Another P5091 derivative, termed P217564 has been generated and shown to impair the suppressive functions of Foxp3^+^ Treg cells in a Tip60-dependent way, thereby enhancing the antitumor immune response *in vivo* (Wang et al., 2016a). Molecular mechanism studies have indicated that P217564 treatment promotes the ubiquitination and degradation of Foxp3 and Tip60, which play essential roles in maintaining the functions of Treg cells. P217564 has also been shown to inhibit tumor growth in syngeneic mice by improving the host antitumor immune responses (Wang et al., 2016b). Of note, the Teff cells from P217564-treated mice have exhibited resistance to the suppression by Treg cells, which suggests that the USP7 inhibitor does not abrogate the functions of Teff cells *in vivo* (Wang et al., 2016a). Further studies have indicated that P217564 is a USP7/USP47 dual inhibitor with IC_50_ values of 0.48 and 0.66 μM, respectively (Wang et al., 2017a). Different from P5091, P217564 directly targets the catalytic core domain of USP7 and forms a covalent adduct with Cys223 on USP7, consequently inducing the inactivation of USP7 and the degradation of USP7 substrates, including MDM2, Foxp3, and Tip60 (Wang et al., 2017a).

### Inhibiting USP7 Activity via Non-covalent Binding

Thanks to the advances in understanding the crystal structures of USP7, USP7-ligand complexes, and the functional domains, especially the catalytic core (Hu et al., 2002), several small-molecule inhibitors have been designed and developed to non-covalently bind to USP7 and selectively inhibit its deubiquitinating activity, including but not limited to the thiazole derivatives C7 and C19 (Chen et al., 2017), XL188 (Lamberto et al., 2017), FT671 (Turnbull et al., 2017), CDDO-Me (Qin et al., 2016), GNE-6640 (Kategaya et al., 2017), GNE-6776 (Kategaya et al., 2017), and compounds 1, 2, 4, and 5 (Gavory et al., 2018).

Some of these inhibitors have been designed to target the catalytic domain of USP7 via non-covalent binding and inhibit its deubiquitinating activity. Chen et al. (2017) have designed and synthesized a new class of thiazole derivatives based on the chemical structure of P22077 and the crystal structure of USP7. The lead compounds C7 and C19 have been found to directly bind to the ubiquitin-binding site of USP7 and inhibit its activity with IC_50_ values of 0.67 and 1.35 μM, respectively (Chen et al., 2017). Both compounds have exhibited anti-proliferation activity in cancer cells *in vitro*, independent of p53 (Chen et al., 2017). Lamberto et al. (2017) have performed a structure-based design and developed a non-covalent-binding USP7 inhibitor termed XL188, which selectively binds to the S4–S5 pocket of USP7 (*K*_*D*_ = 104 nM) and potently inhibits its enzymatic activity (IC_50_ = 90 nM). XL188 has also been found to accelerate MDM2 protein degradation and increase the accumulation of p53 and p21 (Lamberto et al., 2017). However, the *in vivo* efficacy and safety profiles of these inhibitors have not been examined yet.

Turnbull et al. (2017) have recently characterized a non-covalent inhibitor FT671, which binds to the USP7 catalytic domain with a *K*_*D*_ of 65 nM and inhibits USP7 activity with an IC_50_ of 69 nM. FT671 has also been found to decrease the MDM2 level and increase the p53 level in a concentration-dependent manner, thereby blocking the proliferation of MM.1S cells (IC_50_ = 33 nM) *in vitro* and inhibiting the growth of MM.1S xenograft tumors *in vivo* (Turnbull et al., 2017). In addition, a synthetic triterpenoid CDDO-Me has recently been characterized as a USP7 inhibitor using the Ub-AMC protease assay (Qin et al., 2016). Qin et al. (2016) have demonstrated that CDDO-Me directly binds to USP7, markedly inhibits its activity (IC_50_ = 14.08 μM), and induces the degradation of MDM2, MDMX, and UHRF1. CDDO-Me has also shown anticancer activity in ovarian cancer cells *in vitro* and in HO8910 and SKOV3 xenograft models *in vivo*.

Several non-covalent-binding inhibitors have been designed to attenuate the USP7-ubiquitin binding for inhibiting its deubiquitinating activity. Kategaya et al. (2017) have recently developed two selective USP7 inhibitors GNE-6640 and GNE-6776 using nuclear magnetic resonance-based screening and structure-based design. Both compounds non-covalently interact with and cleave ubiquitin moieties harboring free K48 side chains, thereby competitively inhibiting the binding of ubiquitin to USP7 and destabilizing its substrates, especially MDM2 (Kategaya et al., 2017). GNE-6640 and GNE-6776 have also been shown to inhibit the viability of cancer cells and enhance the anticancer activity of chemotherapeutics and targeted agents *in vitro* and delay the EOL-1 xenograft tumor growth *in vivo* (Kategaya et al., 2017).

Gavory et al. (2018) have recently identified a new class of non-covalent and reversible USP7 inhibitors. By employing fragment-based methods as well as combining structural features of published USP7 inhibitors, Gavory et al. (2018) have designed and synthesized a chemically stable and reversible USP7 binder, compound 1 (IC_50_ = 13.1 μM) with high water solubility and without redox liabilities. By introducing a chiral methyl group (*R* stereochemistry) at the benzylic position of the phenethylamide chain, they have further obtained a sub-micromolar USP7 inhibitor, compound 2 (IC_50_ = 0.3 μM) (Gavory et al., 2018). The non-covalent binding of compound 2 to USP7 has been confirmed by the X-ray crystal structure of their complex. The key amino acid residues with conformational changes, including Phe409, Gly458, Gly463, and His461 have been disclosed, indicating a novel binding site that is situated 5.5 Å from the previously reported catalytic cysteine. Further optimization of compound 2 has led to the discovery of highly potent and selective allosteric USP7 inhibitors, compound 4 (IC_50_ = 6.0 nM) and compound 5 (IC_50_ = 22.0 nM) (Gavory et al., 2018). The co-crystal structure of compound 5 and USP7 complex is highly similar to the USP7-compound 2 complex. Besides, compound 4 has been found to tightly bind to USP7 (*K*_*D*_ = 2.0 nM) and non-competitively inhibit its activity. Compound 4 has also been shown to decrease the MDM2 level and increase the levels of p53 and p21 *in vitro*. In comparison to the clinically relevant MDM2 inhibitors, compound 4 has equal or superior anticancer activity in acute lymphoblastic leukemia cell line RS4;11 and prostate cancer cell line LNCaP, which are hypersensitive to USP7 inhibition (Gavory et al., 2018).

### Directly Inhibiting USP7-MDM2 Binding

Zhang et al. (2010) have recently characterized berberine, a natural product isolated from Chinese herbal medicine as a novel USP7 inhibitor with a distinct molecular mechanism. Berberine has been shown to disrupt the MDM2-DAXX-USP7 complex and promote MDM2 self-ubiquitination and degradation. It has also been observed that berberine induces cytotoxicity and apoptosis in acute lymphoblastic leukemia (ALL) cells containing wild-type p53 and overexpressed MDM2 (Zhang et al., 2010). Considering that USP7 binds to MDM2 via the TRAF domain instead of the catalytic core domain, berberine may block the USP7-MDM2 binding via interacting with the TRAF domain. However, the binding mechanisms should be further investigated and may be used to develop specific USP7 inhibitors, whose molecular mechanisms are distinct from the current USP7 inhibitors.

## Conclusion and Future Directions

The MDM2/MDMX-p53 circuitry plays a pivotal role in cancer cell proliferation, cell cycle progression, apoptosis, and senescence (Karni-Schmidt et al., 2016; Wang et al., 2019b), while USP7 is a critical regulator of this circuitry and tightly controls the stabilities of these proteins, thereby contributing to cancer initiation, progression, and metastasis (Bhattacharya et al., 2018; Rawat et al., 2019). Many MDM2/MDMX inhibitors have been developed for cancer therapy (Qin et al., 2015; Wang et al., 2018a, b, 2019b; de Oliveira Ribeiro et al., 2020), and some of them are in clinical trials (Liu et al., 2019b; Rafal et al., 2019). Most of the MDM2/MDMX inhibitors are designed to target the MDM2/p53 and MDMX/p53 interactions, and wild-type p53 is critical for their anticancer activity (Liu et al., 2019b; Rafal et al., 2019). However, p53 mutation and inactivation and MDM2/MDMX overexpression and amplification are frequently observed in various types of human cancer, and the current MDM2/MDMX inhibitors have low or no efficacy in patients with these cancers (Bohlman and Manfredi, 2014; Wang et al., 2014a, b). Therefore, it is urgently needed to find a new strategy for developing effective and safe therapeutics for such types of cancer.

USP7 directly interacts with MDM2 and MDMX, regardless of p53 status. The current USP7 inhibitors have been shown to promote the ubiquitination and degradation of MDM2 and MDMX, independent of p53. Consistently, these USP7 inhibitors have shown anticancer activity in cancer cells with various p53 statuses *in vitro* and *in vivo*. Therefore, targeting the USP7-MDM2/MDMX-p53 network is a promising strategy to develop novel targeted therapy for human cancers, especially for those with mutant p53 and overexpressed MDM2 and MDMX. Currently, almost all the USP7 inhibitors have been developed to directly inhibit its deubiquitinating activity with or without the binding to its catalytic core. However, some of the USP7 substrates function as tumor suppressors, such as p53 and PTEN. Directly inhibiting the enzymatic activity of USP7 with or without binding to the catalytic domain by small-molecule inhibitors may cause the degradation of all the substrates of USP7, regardless of tumor suppressors (e.g., p53 and PTEN) and oncoproteins (e.g., MDM2 and MDMX), which may cause adverse effects in cancer patients.

Inhibiting the expression of USP7 has been considered as a strategy to develop USP7 inhibitors. However, it has been reported that the down-regulation of USP7 expression also leads to the degradation of the p53 tumor suppressor (Tavana and Gu, 2017). Also, small molecules developed to inhibit the expression of molecular targets have been frequently found to have low specificity and selectivity (Qin et al., 2019a, b; Dong et al., 2020). The characterization of berberine as a USP7-MDM2 binding inhibitor provides novel strategies to develop specific inhibitors that selectively promote the degradation of oncoproteins. It is speculated that berberine binds to the TRAF domain of USP7 and blocks the USP7-MDM2 binding, consequently inhibiting USP7-mediated MDM2 deubiquitination and promoting its proteasomal degradation. However, the detailed binding mechanism is still not clear yet. Importantly, it has been reported that the MDM2-USP7 binding is much stronger than the p53-USP7 binding (Hu et al., 2006; Sheng et al., 2006), which provides a rationale for developing selective inhibitors of MDM2-USP7 interaction without affecting the deubiquitinating effects of USP7 on p53. Mechanistically, the small molecules that mimic the P/AxxS motifs and competitively block the bindings of USP7 to MDM2/MDMX may specifically and selectively destabilize MDM2 and MDMX, without affecting other proteins. Nevertheless, this new targeting strategy should be extensively investigated in the future.

## Author Contributions

J-JQ, X-DC, and W-DZ conceptualized the manuscript. S-MQ, GC, X-DC, ZX, BX, and J-JQ collected the literature, wrote the manuscript, and made the figures. J-JQ edited and made significant revisions to the manuscript. All authors read and approved the final manuscript.

## Conflict of Interest

The authors declare that the research was conducted in the absence of any commercial or financial relationships that could be construed as a potential conflict of interest.
